# Targeted plasma lipidomics in non-metastatic colorectal cancer, metastatic colorectal cancer, and healthy controls: an exploratory matched-triplet study

**DOI:** 10.3389/fonc.2026.1917544

**Published:** 2026-08-10

**Authors:** Xiaodong Wang, Huilin Liu, Ke Yin, Guanyi Liao, Ping Ni, Jinjun Guo

**Affiliations:** 1 Department of Gastroenterology, Bishan Hospital of Chongqing Medical University, Chongqing, China; 2 Chongqing Key Laboratory of Precision Medicine for Digestive System Tumors, Bishan Hospital of Chongqing Medical University, Chongqing, China; 3 Department of Radiology, Bishan Hospital of Chongqing Medical University, Chongqing, China

**Keywords:** colorectal cancer, metastatic colorectal cancer, phosphatidylcholine, propensity score matching, sphingolipid, targeted lipidomics

## Abstract

**Background:**

Systemic lipid remodeling accompanies colorectal cancer (CRC), but whether circulating lipid alterations specifically distinguish metastatic from non-metastatic disease remains uncertain.

**Methods:**

This single-center prospective cross-sectional study enrolled newly diagnosed patients with non-metastatic CRC (nmCRC), metastatic CRC (mCRC), and healthy controls (HC) between September and December 2024. Two separate 1:1 nearest-neighbor propensity score matches used the same mCRC cohort as the anchor and histories of diabetes and hyperlipidemia as covariates. Matching was performed without replacement using a caliper of 0.2 on the raw propensity-score scale. Participant-level linkage generated 20 matched triplets. Fasting plasma samples underwent quantitative targeted lipidomic profiling. Primary lipid comparisons used matched-pair tests with comparison-specific Benjamini-Hochberg correction, and age- and sex-adjusted sensitivity analyses evaluated residual baseline imbalance.

**Results:**

Among 1,459 quantified lipids, 72 nominal differential lipids were identified in mCRC versus nmCRC, 186 in nmCRC versus HC, and 222 in mCRC versus HC using paired raw P<0.05. No lipid remained significant after false discovery rate correction in mCRC versus nmCRC, whereas 12 and 30 lipids remained significant in nmCRC versus HC and mCRC versus HC, respectively. After age and sex adjustment, the corresponding FDR-significant counts were 0, 10, and 9. Eight nominal candidates were shared across all three primary comparisons: BMP(14:0_20:0), BMP(14:0_20:1), Cer(d18:1/26:1), PC(18:2_18:2), PC(18:3_20:1), PC(18:3_20:4), PC(20:0_22:5), and PI(20:0_20:4).

**Conclusion:**

Plasma lipidomic differences were most reproducible between patients with CRC and healthy controls, particularly between mCRC and HC. Lipid differences between mCRC and nmCRC remained exploratory and hypothesis-generating. These findings indicate systemic lipid remodeling in CRC but do not establish metastasis-specific plasma biomarkers.

## Introduction

Colorectal cancer (CRC) remains one of the most commonly diagnosed malignancies worldwide and continues to impose a substantial mortality burden (1). Recent population-based statistics also demonstrate a changing CRC burden across age groups (2). Distant metastasis is a major determinant of treatment selection and long-term prognosis. Patients presenting with synchronous metastases frequently enter systemic treatment pathways, and clinical decisions depend on metastatic site, tumor burden, biological aggressiveness, and host condition (3, 4). Molecular characterization of circulating metabolic alterations may therefore complement conventional clinical assessment, although biomarkers intended to distinguish metastatic from non-metastatic disease require particularly rigorous validation.

Lipids are structural components of cellular membranes, energy-storage molecules, and regulators of receptor signaling, oxidative stress, immune function, and regulated cell-death programs (5, 6). CRC is accompanied by altered lipid uptake, synthesis, oxidation, and phospholipid remodeling, while metabolic interactions between tumor and immune cells may influence invasion, treatment resistance, and metastatic niche formation (5). Lipidomics provides molecular characterization of lipid classes and species and has been investigated for CRC detection and disease characterization. Recurrent observations include changes in choline phospholipids, ether lipids, and sphingolipids, although reported signatures vary across analytical platforms and patient populations (7–9).

Clinical lipidomic profiles are sensitive to pre-analytical handling, including patient preparation, sample collection, processing, storage, and freeze-thaw conditions (10). Good-practice recommendations emphasize transparent sample processing and internal-standard monitoring (11). Dedicated reporting checklists support reproducible documentation of lipidomics experiments (12, 13), while updated LIPID MAPS guidance provides standardized classification and structural notation (14). These issues are especially important in small studies in which nominally significant lipid signals may not remain significant after false discovery rate (FDR) correction.

In this study, quantitative targeted plasma lipidomics was used to compare patients with nmCRC, patients with mCRC, and HC participants within a verified linked matched-triplet cohort. The objectives were to characterize systemic lipid alterations associated with CRC, assess whether circulating lipids distinguished mCRC from nmCRC after multiple-testing correction, and evaluate the influence of residual age and sex imbalance.

## Materials and methods

### Study design and participants

**Figure 1:**
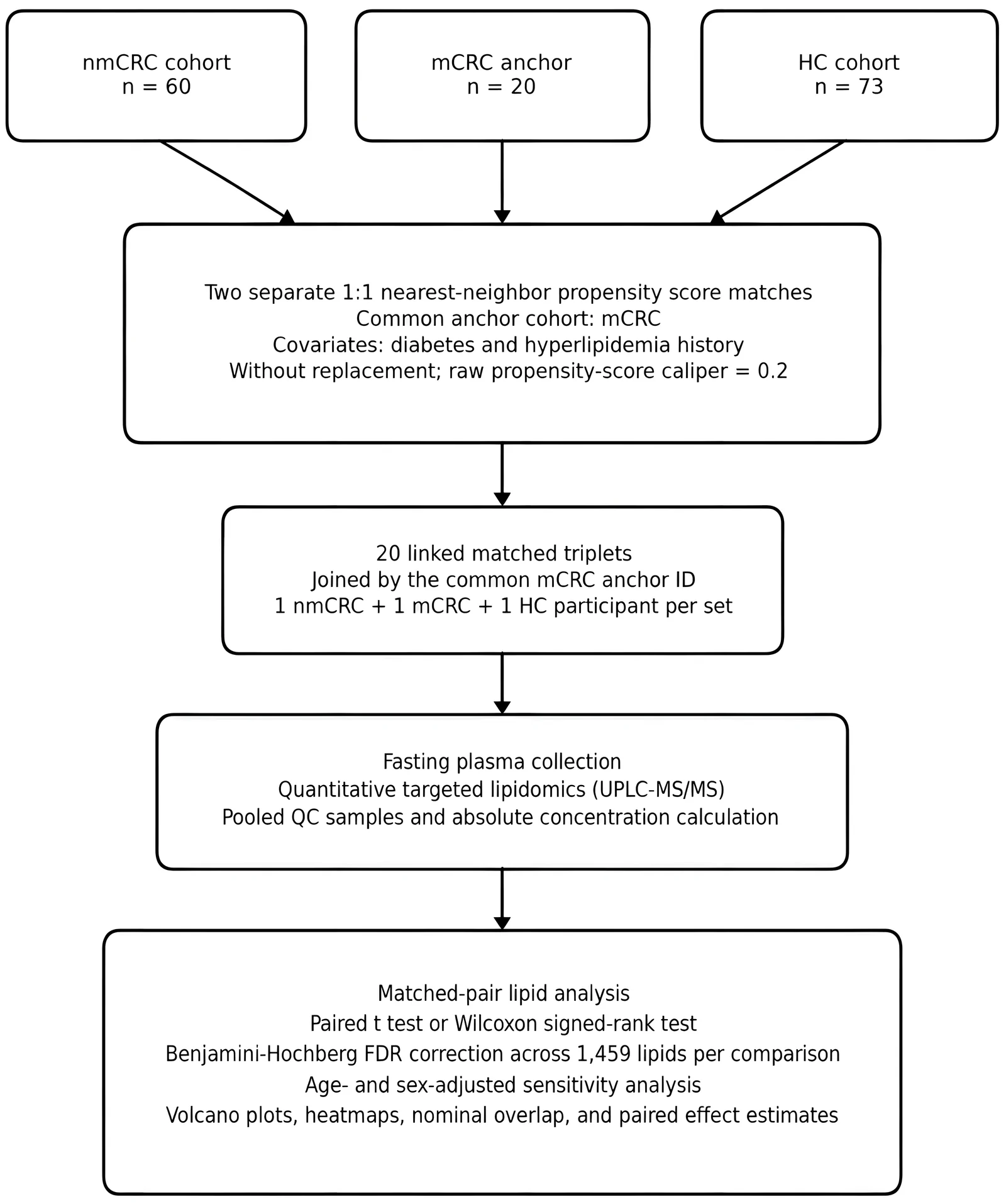
Study workflow and linked matched-triplet construction.

This single-center prospective cross-sectional observational study was reported in accordance with the STROBE statement (15). Patients with CRC and healthy controls were prospectively recruited at Bishan Hospital of Chongqing Medical University between September and December 2024. The initial cohorts comprised 60 newly diagnosed patients with nmCRC, 20 newly diagnosed patients with mCRC, and 73 HC participants (**Figure 1**). The study was approved by the Institutional Ethics Review Committee of Bishan Hospital of Chongqing Medical University (approval No. cqbyklyy-20240918-24) and was conducted in accordance with the Declaration of Helsinki. Written informed consent was obtained from all participants.

Propensity score matching (PSM) was performed to reduce imbalance in two prespecified metabolic comorbidities: history of diabetes and history of hyperlipidemia. Residual age and sex imbalance was instead quantified using standardized mean differences and addressed through age- and sex-adjusted lipid-level sensitivity analyses. The same 20 mCRC participants served as the common anchor cohort in two separate 1:1 nearest-neighbor matching procedures, namely nmCRC versus mCRC and HC versus mCRC. Matching was performed without replacement using a caliper width of 0.2 on the raw propensity-score scale. For each mCRC anchor, the selected nmCRC participant and HC participant from the two matching outputs were linked using the common mCRC anchor identifier. This generated 20 linked matched triplets, each containing one nmCRC participant, one mCRC participant, and one HC participant. Participant-level auditing confirmed that no participant appeared in more than one final matched set. Anonymized matched-set composition is provided in **Supplementary Table 1**.

### Sample processing and targeted lipidomics

A quantitative targeted lipidomics platform was used for absolute quantification of fasting plasma samples. The workflow included sample storage, lipid extraction, ultra-performance liquid chromatography-tandem mass spectrometry (UPLC-MS/MS), quality control, and concentration calculation. Plasma samples were stored at -80 °C immediately after collection and were thawed on ice and vortex-mixed before analysis.

For lipid extraction, 50 µL plasma was mixed with 200 µL methanol and vortexed for 20 s, followed by the addition of 750 µL methyl tert-butyl ether containing internal standards and vortexing for 40 s. After 10 min ultrasonication and 30 min standing at room temperature, 200 µL MS-grade water was added. The mixture was vortexed for 20 s, allowed to stand for 10 min, and centrifuged at 12,000 rpm for 15 min at 4 °C. A 700-µL aliquot of supernatant was transferred and vacuum-dried at 25 °C. The residue was reconstituted in 200 µL dichloromethane:methanol (1:1, v/v), vortexed, ultrasonicated in an ice-water bath for 10 min, and centrifuged again before transfer to an autosampler vial.

### UPLC-MS/MS conditions

Data were acquired in positive and negative electrospray ionization modes using multiple reaction monitoring. Reversed-phase and hydrophilic interaction chromatography systems were used to cover lipids with different polarities.

For reversed-phase analysis, mobile phase A consisted of H2O:acetonitrile:methanol (1:1:1, v/v/v) containing 7 mmol/L ammonium acetate, and mobile phase B consisted of isopropanol containing 7 mmol/L ammonium acetate. Separation was performed on a Kinetex C18 column (2.1 x 100 mm, 2.6 µm; Phenomenex) at 0.35 mL/min. The column temperature was 45 °C, the autosampler temperature was 10 °C, and the injection volume was 1 µL.

For hydrophilic interaction chromatography, mobile phase A consisted of H2O:acetonitrile (1:1, v/v) containing 2 mmol/L ammonium acetate, and mobile phase B consisted of dichloromethane:acetonitrile (1:13, v/v) containing 2 mmol/L ammonium acetate. Separation was performed on a Luna NH2 column (2.0 x 100 mm, 3 µm; Phenomenex) at 0.7 mL/min. The column temperature was 40 °C, the autosampler temperature was 10 °C, and the injection volume was 1 µL.

The chromatographic platform consisted of an Agilent 1290 Infinity II UHPLC system coupled to a SCIEX Triple Quad 7500+ mass spectrometer. IonSpray Voltage was set at +3000/-3000 V, Curtain Gas at 35 psi, Ion Source Gas 1 at 40 psi, Ion Source Gas 2 at 70 psi, and source temperature at 325 °C in positive mode and 550 °C in negative mode. Data acquisition and quantification were performed using SCIEX OS version 3.3.1, with batch processing supported by BIOTREE BioBud version 2.0.4.

### Quality control and concentration calculation

Process blanks and pooled quality-control (QC) samples were used to monitor analytical stability. One pooled QC sample, generated by combining equal aliquots from participant samples, was inserted after every 10 study samples. All study samples were processed and analyzed within the same batch, and run order was interleaved across groups.

System stability was evaluated using retention-time and response stability of internal standards. Retention-time deviations were controlled within ±12 s, QC correlation coefficients were required to exceed 0.8, and relative standard deviations of internal-standard responses in QC samples were controlled at ≤20%. Absolute lipid concentrations were calculated from the ratio of analyte peak area to the corresponding class-specific internal-standard peak area and were expressed as nmol/L. Concentration was calculated as measured concentration x extraction volume x dilution factor/sample volume x concentration factor.

### Statistical analysis

PSM was performed separately for nmCRC versus mCRC and HC versus mCRC using diabetes history and hyperlipidemia history as the matching covariates. Matching was performed without replacement using a caliper width of 0.2 on the raw propensity-score scale. Covariate balance was evaluated using absolute standardized mean differences (SMDs) for all available baseline variables, including age, sex, diabetes history, and hyperlipidemia history; an absolute SMD <0.10 was considered indicative of adequate balance. Postmatching covariate balance was displayed in **Supplementary Figure 1**. Because the final dataset consisted of 20 linked matched triplets, overall three-group comparisons used the Friedman test for continuous variables and Cochran’s Q test for categorical variables. Pairwise comparisons used paired t tests, Wilcoxon signed-rank tests, or McNemar tests, as appropriate.

For each lipid, normality of the within-pair concentration differences was assessed using the Shapiro-Wilk test. A paired t test was used when paired differences were approximately normally distributed; otherwise, the Wilcoxon signed-rank test was applied. Comparison directions were standardized as mCRC/nmCRC, nmCRC/HC, and mCRC/HC. Fold change was calculated as the arithmetic mean concentration in the numerator group divided by that in the denominator group. Raw P values for all 1,459 lipids were independently adjusted using the Benjamini-Hochberg procedure within each comparison.

Lipids with paired raw P<0.05 were classified as nominal differential signals for exploratory visualization and overlap analysis, whereas lipids with q<0.05 were considered statistically robust. Candidate-set overlap was descriptive and was not interpreted as confirmatory evidence. Volcano plots and row-standardized heatmaps were used for lipid-level visualization. No supervised multivariable classification model was used for inferential analysis or nominal-signal selection.

Residual age and sex imbalance was evaluated in a sensitivity analysis of within-pair log2 concentration ratios using age and sex differences as covariates and HC3 heteroskedasticity-consistent standard errors. Benjamini-Hochberg correction was applied separately to the 1,459 adjusted P values in each comparison. For shared nominal candidates, paired effects were summarized using arithmetic fold change, mean within-pair log2 ratio with 95% confidence interval, and matched-pairs rank-biserial correlation. **Supplementary Table 2** provides complete primary and adjusted lipid-level results.

## Results

### Matched cohort and clinical characteristics

After matching and participant-level linkage, 60 participants were included as 20 matched triplets. Diabetes history and hyperlipidemia history were completely balanced in both anchor-group comparisons (absolute SMDs<0.001). Residual imbalance remained for age (absolute SMD, 0.821 for mCRC versus nmCRC and 0.979 for mCRC versus HC) and sex (0.643 and 0.523, respectively). These imbalances were addressed using age- and sex-adjusted lipid-level sensitivity analyses. Complete baseline SMDs are reported in **Table 1** and displayed in **Supplementary Figure 1**.

**Table 1:** Baseline characteristics and standardized mean differences in the linked matched-triplet cohort.

Variable	nmCRC (n=20)	mCRC (n=20)	HC (n=20)	Overall P value	|SMD| mCRC vs nmCRC	|SMD| mCRC vs HC
Age, years	60.5 (54.3-69.0)	72.0 (57.8-81.0)	58.0 (53.5-66.5)	0.081	0.821	0.979
Male sex, n (%)	15 (75.0)	9 (45.0)	14 (70.0)	0.144	0.643	0.523
Diabetes history, n (%)	4 (20.0)	4 (20.0)	4 (20.0)	1.000	<0.001	<0.001
Hyperlipidemia history, n (%)	6 (30.0)	6 (30.0)	6 (30.0)	1.000	<0.001	<0.001

Data are median (interquartile range) or n (%). Absolute SMD <0.10 indicated adequate balance. Diabetes and hyperlipidemia history were included in the propensity score models; age and sex were not included and showed residual imbalance. Overall P values were calculated using the Friedman test for age and Cochran’s Q test for sex.

Among the 20 patients with mCRC, 9 (45%) had liver metastases, 5 (25%) had lung metastases, and 6 (30%) had peritoneal metastases. Abnormal CEA was observed in 3 of 20 patients with nmCRC and 12 of 20 patients with mCRC, whereas abnormal CA19-9 was observed in 1 of 20 and 8 of 20 patients, respectively (**Table 2**).

**Table 2:** Tumor-related clinical characteristics in the matched CRC groups.

Variable	nmCRC (n=20)	mCRC (n=20)
Metastatic site	Not applicable	Liver 9 (45%); lung 5 (25%); peritoneum 6 (30%)
CEA abnormal, n (%)	3 (15%)	12 (60%)
CA19-9 abnormal, n (%)	1 (5%)	8 (40%)

Tumor marker distributions are reported descriptively because participant-level paired marker data were not part of the lipidomic reanalysis dataset.

### Global lipidomic patterns and overview of differential lipids

**Figure 2:**
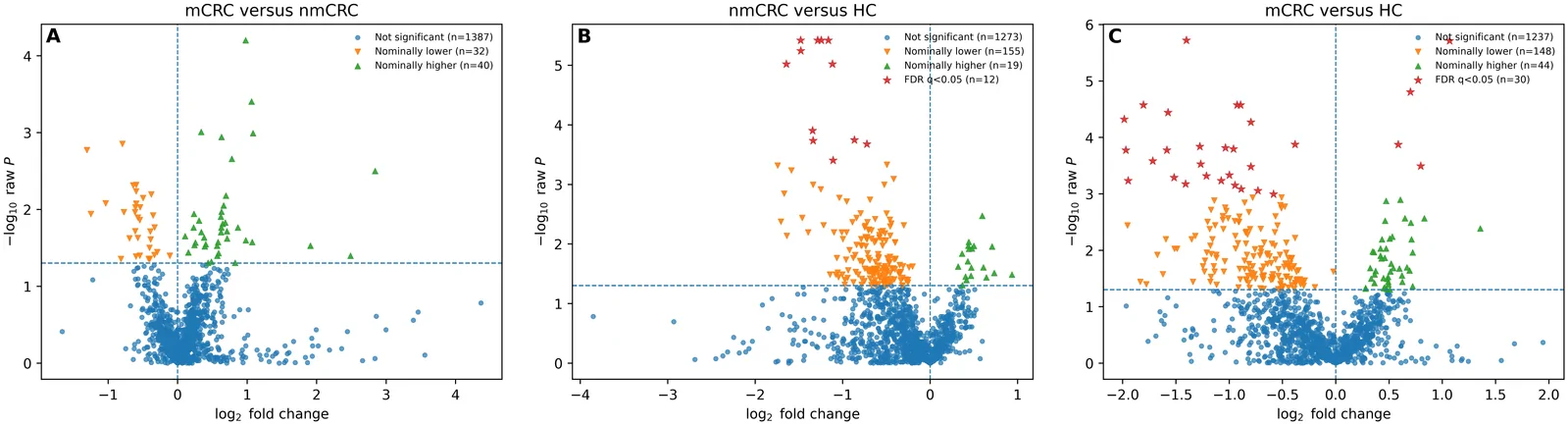
Volcano plots of paired plasma lipid comparisons. **(A)** mCRC versus nmCRC. **(B)** nmCRC versus HC. **(C)** mCRC versus HC. Lipids with paired raw P<0.05 are displayed as nominal differential signals. Higher and lower counts refer to the numerator group. FDR-adjusted q<0.05 was required for statistically robust inference.

**Figure 3:**
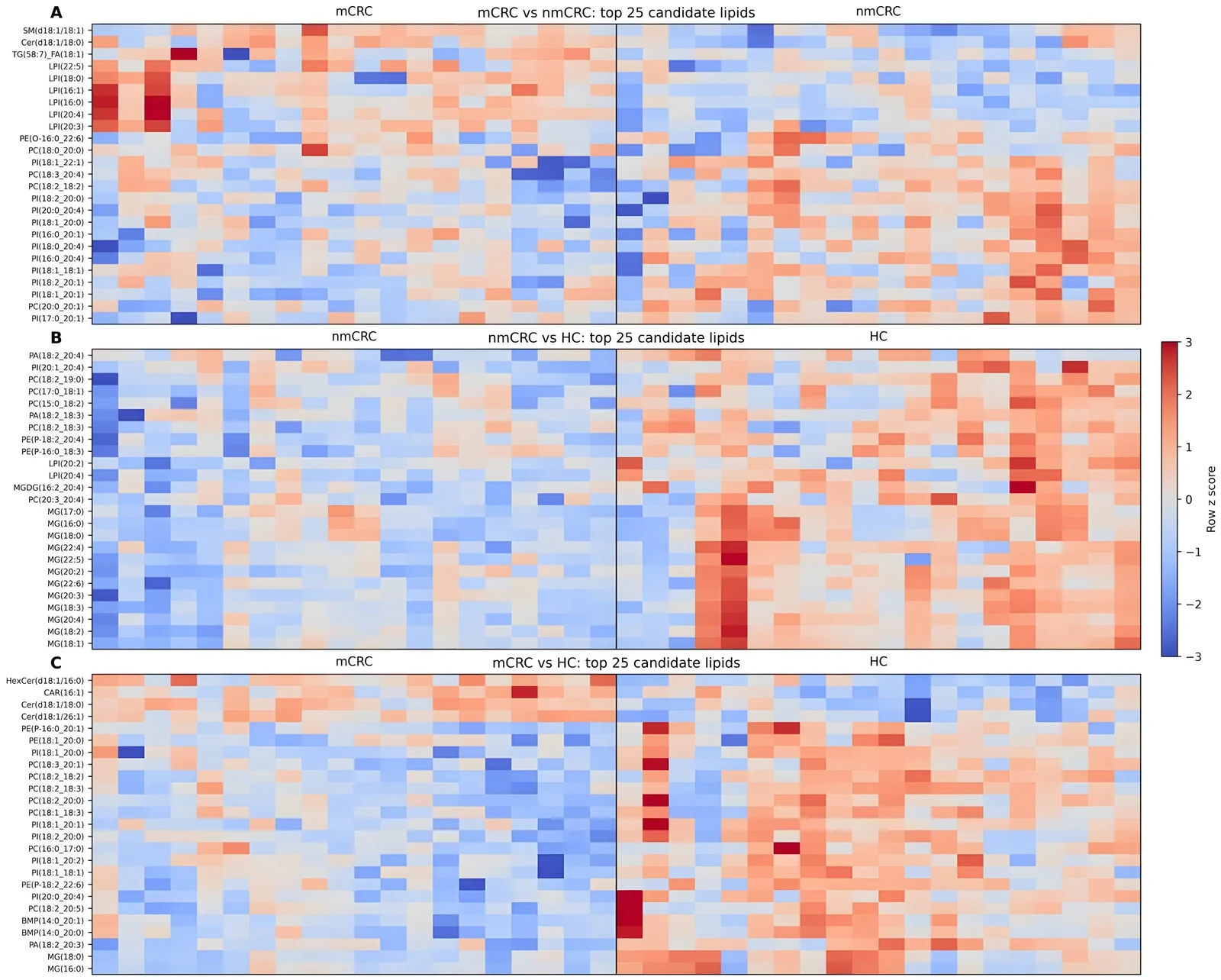
Heatmaps of the top-ranked nominal lipid differences in the three paired comparisons. Rows represent lipids and columns represent participants. Values are row-standardized z scores.

A total of 1,459 lipid molecules were quantified. Volcano plots summarized the direction and statistical ranking of paired lipid differences, and row-standardized heatmaps displayed participant-level patterns (**Figures 2**, **3**). Because the number of lipid variables substantially exceeded the number of participants, supervised multivariable discrimination was not retained in the inferential framework.

Using paired raw P<0.05 as the nominal threshold, 72 lipids were identified in mCRC versus nmCRC, including 40 higher and 32 lower lipids in mCRC. The nmCRC-versus-HC comparison yielded 186 nominal signals, including 19 higher and 167 lower lipids in nmCRC. The mCRC-versus-HC comparison yielded 222 nominal signals, including 48 higher and 174 lower lipids in mCRC (**Table 3**).

**Table 3:** Nominal and FDR-significant lipids after matched-pair analysis.

Comparison	Total lipids	Raw P<0.05	Higher	Lower	BH q<0.05
mCRC vs nmCRC	1,459	72	40	32	0
nmCRC vs HC	1,459	186	19	167	12
mCRC vs HC	1,459	222	48	174	30

Nominal differential lipids were defined by paired raw P<0.05. Higher and lower refer to the numerator group in the stated comparison. BH-adjusted q<0.05 was required for statistically robust inference.

### FDR-robust lipid differences

No lipid remained significant after FDR correction in the primary mCRC-versus-nmCRC comparison. Therefore, all lipid-level findings distinguishing metastatic from non-metastatic CRC were retained only as exploratory, hypothesis-generating signals.

In contrast, 12 lipids remained significant after FDR correction in nmCRC versus HC. These comprised eight monoacylglycerols, two lysophosphatidylinositols, one phosphatidic acid, and one plasmenyl phosphatidylethanolamine, and all showed lower concentrations in nmCRC than in HC. The full results are provided in **Supplementary Table 2**.

The mCRC-versus-HC comparison yielded 30 FDR-significant lipids in the primary paired analysis (**Table 4**). After adjustment for age and sex, 9 of these lipids remained FDR significant. In the nmCRC-versus-HC comparison, 10 of the 12 primary FDR-significant lipids remained significant after adjustment, whereas no lipid reached adjusted q<0.05 in mCRC versus nmCRC. Complete adjusted results are provided in **Supplementary Table 2**.

**Table 4:** Lipids significant between mCRC and HC after FDR correction in the primary paired analysis.

Lipid	Subclass	FC (mCRC/HC)	Raw P	BH q
PI(20:0_20:4)	PI	0.378	1.91e-06	0.0014
Cer(d18:1/18:0)	Cer_NS	2.098	1.95e-06	0.0014
Cer(d18:1/26:1)	Cer_NS	1.625	1.57e-05	0.0065
PC(18:2_20:0)	PC	0.538	2.67e-05	0.0065
PI(18:1_20:1)	PI	0.286	2.67e-05	0.0065
PI(18:1_20:2)	PI	0.527	2.67e-05	0.0065
PC(18:3_20:1)	PC	0.336	3.62e-05	0.0076
MG(18:0)	MG	0.253	4.77e-05	0.0087
PA(18:2_20:3)	PA	0.576	5.4e-05	0.0088
PC(16:0_17:0)	PC	0.768	0.000134	0.0153
HexCer(d18:1/16:0)	HexCer_NS	1.502	0.000134	0.0153
PI(18:1_20:0)	PI	0.413	0.000145	0.0153
PC(18:1_18:3)	PC	0.488	0.000153	0.0153
PC(18:2_18:2)	PC	0.514	0.000161	0.0153
BMP(14:0_20:1)	BMP	0.255	0.000168	0.0153
PC(18:2_20:5)	PC	0.333	0.000168	0.0153
BMP(14:0_20:0)	BMP	0.304	0.000261	0.0224
PC(18:2_18:3)	PC	0.415	0.000297	0.0241
CAR(16:1)	CAR	1.738	0.000322	0.0241
PI(18:1_18:1)	PI	0.576	0.000331	0.0241
PE(P-18:2_22:6)	PE-P	0.500	0.000467	0.0320
PE(P-16:0_20:1)	PE-P	0.431	0.000483	0.0320
PI(18:2_20:0)	PI	0.349	0.000514	0.0326
MG(16:0)	MG	0.259	0.000586	0.0342
PE(18:1_20:0)	PE	0.475	0.000586	0.0342
PE(P-18:2_20:4)	PE-P	0.376	0.000667	0.0374
PA(18:2_20:0)	PA	0.519	0.000708	0.0382
PE(P-16:1_20:4)	PE-P	0.541	0.000821	0.0428
PE(P-16:0_16:0)	PE-P	0.603	0.000879	0.0442
PC(20:0_20:4)	PC	0.667	0.00102	0.0494

FC was calculated as mean concentration in mCRC divided by mean concentration in HC. The selected paired test and full group summaries are provided in **Supplementary Table 2**.

### Lipid-class composition of candidate signals

In the mCRC-versus-nmCRC nominal set, PI was the most frequent subclass, followed by PC and LPI. Smaller contributions were observed from carnitines, sphingomyelins, BMP, MGDG, PA, PE, PE-P, ceramides, and other classes. This heterogeneous distribution did not yield an FDR-robust metastatic-state signature.

The nmCRC-versus-HC nominal set was dominated by PC and PE-P, with additional MG, PA, BMP, PI, TG, LPC, and LPI species. The mCRC-versus-HC set was likewise dominated by PC and PE-P but included broader PI, PA, PE, LPC, MG, TG, BMP, and ceramide contributions. The FDR-significant findings and adjusted sensitivity results indicate that the most reproducible differences reflected systemic CRC-associated lipid alterations rather than a validated metastasis-specific signature.

### Shared candidate lipids and paired effect sizes

**Figure 4:**
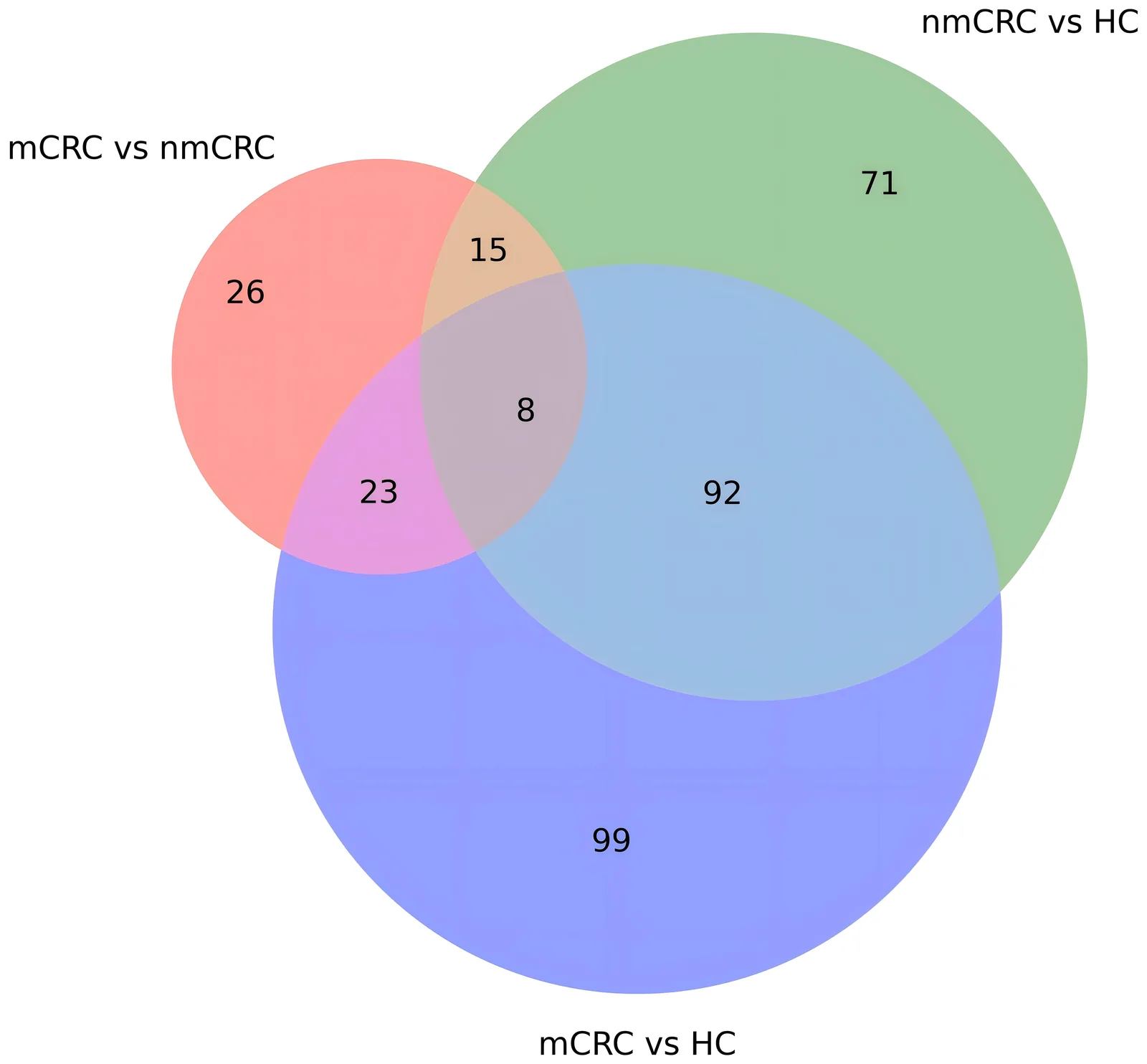
Venn diagram of shared and comparison-specific nominal differential lipids after matched-pair analysis.

Venn analysis showed 26 lipids unique to mCRC versus nmCRC, 71 unique to nmCRC versus HC, and 99 unique to mCRC versus HC. Pairwise-only intersections contained 15, 23, and 92 lipids, respectively, and 8 nominal candidates were shared across all three comparisons (**Figure 4**): BMP(14:0_20:0), BMP(14:0_20:1), Cer(d18:1/26:1), PC(18:2_18:2), PC(18:3_20:1), PC(18:3_20:4), PC(20:0_22:5), and PI(20:0_20:4) (**Table 5**).

**Table 5:** Eight nominal candidate lipids shared across all three pairwise comparisons.

Lipid	Subclass	mCRC vs nmCRC	nmCRC vs HC	mCRC vs HC
BMP(14:0_20:0)	BMP	0.672 (0.0128; 0.665)	0.452 (0.0328; 0.332)	0.304 (0.000261; 0.0224)
BMP(14:0_20:1)	BMP	0.795 (0.0172; 0.696)	0.321 (0.0073; 0.205)	0.255 (0.000168; 0.0153)
Cer(d18:1/26:1)	Cer_NS	1.195 (0.027; 0.771)	1.360 (0.00927; 0.21)	1.625 (1.57e-05; 0.00649)
PC(18:2_18:2)	PC	0.769 (0.00639; 0.665)	0.669 (0.00714; 0.205)	0.514 (0.000161; 0.0153)
PC(18:3_20:1)	PC	0.660 (0.0409; 0.905)	0.509 (0.0484; 0.389)	0.336 (3.62e-05; 0.00755)
PC(18:3_20:4)	PC	0.661 (0.00582; 0.665)	0.672 (0.0353; 0.346)	0.444 (0.00193; 0.0688)
PC(20:0_22:5)	PC	0.926 (0.04; 0.898)	0.794 (0.0296; 0.329)	0.735 (0.0136; 0.177)
PI(20:0_20:4)	PI	0.577 (0.0014; 0.34)	0.656 (0.0266; 0.328)	0.378 (1.91e-06; 0.00142)

Values are fold change (raw P; BH q). Comparison directions are numerator/denominator as stated. Shared membership was defined by raw P<0.05 in all three comparisons and does not represent FDR-confirmed overlap.

Among the eight shared nominal candidates, Cer(d18:1/26:1) was higher in mCRC than in both nmCRC and HC. The two BMP species, four PC species, and PI(20:0_20:4) were lower in mCRC. In the primary mCRC-versus-HC analysis, six of the eight shared candidates reached q<0.05; after age and sex adjustment, only PC(18:3_20:1) and PI(20:0_20:4) remained FDR significant. None reached adjusted q<0.05 in mCRC versus nmCRC (**Table 6**; **Supplementary Table 2**).

**Table 6:** Paired effect estimates for the eight shared nominal candidate lipids.

Lipid	Comparison	FC	BH q	Mean paired log2 ratio (95% CI)	Matched-pairs rank-biserial correlation
BMP(14:0_20:0)	mCRC vs nmCRC	0.672	0.6649	-0.596 (-0.973, -0.219)	-0.705
BMP(14:0_20:0)	mCRC vs HC	0.304	0.0224	-1.560 (-2.261, -0.860)	-0.857
BMP(14:0_20:1)	mCRC vs nmCRC	0.795	0.6963	-0.263 (-0.571, 0.045)	-0.600
BMP(14:0_20:1)	mCRC vs HC	0.255	0.0153	-1.479 (-2.139, -0.820)	-0.876
Cer(d18:1/26:1)	mCRC vs nmCRC	1.195	0.7710	0.289 (0.043, 0.535)	0.524
Cer(d18:1/26:1)	mCRC vs HC	1.625	0.0065	0.746 (0.454, 1.037)	0.943
PC(18:2_18:2)	mCRC vs nmCRC	0.769	0.6649	-0.381 (-0.673, -0.089)	-0.629
PC(18:2_18:2)	mCRC vs HC	0.514	0.0153	-0.926 (-1.358, -0.494)	-0.876
PC(18:3_20:1)	mCRC vs nmCRC	0.660	0.9051	-0.678 (-1.345, -0.010)	-0.610
PC(18:3_20:1)	mCRC vs HC	0.336	0.0076	-1.325 (-1.817, -0.833)	-0.933
PC(18:3_20:4)	mCRC vs nmCRC	0.661	0.6649	-0.812 (-1.270, -0.353)	-0.695
PC(18:3_20:4)	mCRC vs HC	0.444	0.0688	-1.262 (-2.031, -0.493)	-0.714
PC(20:0_22:5)	mCRC vs nmCRC	0.926	0.8976	-0.100 (-0.359, 0.159)	-0.524
PC(20:0_22:5)	mCRC vs HC	0.735	0.1770	-0.398 (-0.749, -0.048)	-0.619
PI(20:0_20:4)	mCRC vs nmCRC	0.577	0.3403	-0.712 (-1.116, -0.309)	-0.829
PI(20:0_20:4)	mCRC vs HC	0.378	0.0014	-1.198 (-1.637, -0.758)	-1.000

Fold changes are arithmetic mean ratios. Mean paired log2 ratios and 95% confidence intervals summarize within-pair effects. Positive effect estimates indicate higher concentrations in the numerator group.

HexCer(d18:1/16:0) was not included in the three-way nominal intersection, but it remained FDR significant in mCRC versus HC in both the primary and age- and sex-adjusted analyses.

## Discussion

Quantitative targeted plasma lipidomics demonstrated broader differences between either CRC group and healthy controls than between metastatic and non-metastatic CRC. No lipid reached FDR significance in mCRC versus nmCRC in either the primary paired analysis or the age- and sex-adjusted sensitivity analysis. Twelve and 30 lipids were FDR significant in the primary nmCRC-versus-HC and mCRC-versus-HC comparisons, of which 10 and 9, respectively, remained significant after adjustment. These findings support systemic lipid remodeling in CRC but do not establish a metastasis-specific circulating signature.

The primary nmCRC-versus-HC profile was characterized predominantly by lower monoacylglycerols, whereas the mCRC-versus-HC profile encompassed a wider range of phospholipid and sphingolipid classes. The marked attenuation of the mCRC-versus-HC robust set after age and sex adjustment indicates that part of the primary difference was attributable to residual baseline imbalance. The remaining adjusted signals may reflect a combination of malignant disease, tumor burden, organ involvement, nutritional status, inflammation, and host metabolic responses.

Cer(d18:1/26:1) was FDR significant in the primary mCRC-versus-HC analysis but did not remain significant after age and sex adjustment. In contrast, HexCer(d18:1/16:0) remained increased and FDR significant in the adjusted analysis. Ceramides and glycosphingolipid derivatives participate in membrane-domain organization, receptor signaling, apoptosis, immune regulation, and therapeutic response (16, 17), and altered ceramide synthase biology has been implicated in CRC (18). Nevertheless, circulating concentrations do not identify the tissue of origin.

Several PC, PI, and BMP species were lower in mCRC in the primary analysis. Among the shared nominal candidates, PC(18:3_20:1) and PI(20:0_20:4) remained FDR significant in the adjusted mCRC-versus-HC analysis, whereas the other shared phospholipids did not. LPCAT1 overexpression provides evidence of altered choline phospholipid remodeling in CRC (19), and broader lipidomics literature supports possible links with membrane organization, vesicle formation, receptor function, and lipid peroxidation (20–23). These mechanisms remain biologically plausible interpretations rather than pathways demonstrated in the present plasma cohort.

Eight lipids were common to all three nominal sets. This overlap may help prioritize species for further study, but it was defined using unadjusted raw P<0.05 and should not be interpreted as statistical confirmation. Only two of the eight remained FDR significant in the adjusted mCRC-versus-HC analysis, and none remained significant in the adjusted mCRC-versus-nmCRC comparison.

The CRC-versus-HC findings are broadly consistent with previous plasma and extracellular-vesicle lipidomics studies reporting phosphatidylcholine, plasmalogen, and sphingolipid alterations (7–9). Direct comparison remains difficult because of differences in sample processing, analytical platforms, fasting status, patient selection, and statistical thresholds. Absolute targeted quantification, pooled QC monitoring, matched-pair testing, multiple-testing correction, and age- and sex-adjusted sensitivity analysis improved internal transparency, but independent external replication remains essential.

Recent original studies provide additional context for the present findings. Serum metabolomic and lipidomic profiling across the colorectal adenoma-carcinoma sequence demonstrated marked disturbances in phosphatidylcholine and triacylglycerol metabolism during CRC development (24). Plasma metabolic and lipidomic analyses also identified distinct molecular profiles between early-onset and late-onset CRC, indicating that circulating lipid signatures may vary according to age-related host characteristics (25). Furthermore, a targeted plasma lipidomic study associated specific circulating lipid species with CRC risk and demonstrated effect modification by common risk factors and environmental exposure, supporting cautious interpretation of lipid signatures obtained from small cross-sectional cohorts (26). At the tissue level, integrated lipidomics, single-cell, and spatial transcriptomic analyses revealed a persistent pro-inflammatory lipid state in colon cancer (27), while recent mechanistic evidence implicated phosphatidylcholine metabolic reprogramming in CRC progression, metastatic behavior, drug resistance, and tumor-associated neutrophil interactions (28). Collectively, these findings support the biological plausibility of the observed phospholipid and sphingolipid alterations but also emphasize that circulating lipid profiles are context-dependent and require independent validation.

Several limitations should be considered. First, this was a single-center exploratory study with only 20 matched triplets. Second, the propensity score models included diabetes and hyperlipidemia history but not age or sex, and substantial residual imbalance remained. BMI, recent weight loss, routine lipid concentrations, albumin, C-reactive protein, comprehensive liver and renal function, and detailed lipid-modifying medication exposure were not uniformly available. Third, 45% of the mCRC group had liver metastases. Fourth, the cross-sectional design precludes causal inference. Finally, no supervised multivariable classification model was retained because the high variable-to-sample ratio created a substantial risk of overfitting; nominal overlap analysis remains exploratory.

Further studies should use larger multicenter cohorts with standardized collection of anthropometric, nutritional, inflammatory, hepatic, renal, and medication variables. Prespecified stratification by metastatic site and tumor burden, together with longitudinal sampling from diagnosis through treatment and progression, will be important for distinguishing disease-associated lipid changes from treatment effects and host metabolic responses. Integration of plasma findings with tumor-tissue and extracellular-vesicle lipidomics may further clarify the biological origin and translational relevance of the candidate lipid species.

## Conclusion

Quantitative plasma lipidomics identified systemic lipid differences between CRC and healthy controls, but the extent of the mCRC-versus-HC signal was substantially attenuated after age and sex adjustment. No lipid remained FDR significant in mCRC versus nmCRC in either the primary or adjusted analysis. The eight shared nominal candidates should therefore be considered hypothesis-generating, and the present data do not establish metastasis-specific plasma biomarkers.

## Data Availability

The raw data supporting the conclusions of this article will be made available by the authors, without undue reservation.

## Supplementary material

Supplementary Table 1 Anonymized participant composition, baseline characteristics, and construction details of the 20 linked matched triplets.

Supplementary Table 2 Complete primary matched-pair and age- and sex-adjusted lipid-level results for all 1,459 lipids across the three pairwise comparisons.

Supplementary Figure 1 Postmatching covariate balance in the two propensity score matches. **(A)** mCRC versus nmCRC. **(B)** mCRC versus HC. Absolute standardized mean differences are displayed for age, sex, diabetes history, and hyperlipidemia history. The dashed vertical line indicates the prespecified balance threshold of 0.10.
